# COVID-19 and liver diseases

**DOI:** 10.1186/s43066-022-00202-2

**Published:** 2022-07-21

**Authors:** Maged T. Elghannam, Moataz H. Hassanien, Yosry A. Ameen, Gamal M. ELattar, Ahmed A. ELRay, Emad A. Turky, Mohammed D. ELTalkawy

**Affiliations:** Theodor Bilharz Research Institute, Giza, Egypt

**Keywords:** Coronaviruses, SARS-CoV-2, Liver diseases, Hepatocellular carcinoma, Autoimmune liver disease, Liver transplantation, COVID-19 vaccination

## Abstract

Coronavirus causes an outbreak of viral pneumonia that spread throughout the world. Liver injury is becoming more widely recognized as a component of the clinical picture of COVID-19 infection. Hepatitis with serum ALT elevation has been reported in up to half of patients. Patients with CLD were at a higher risk of decompensation with liver failure, hospitalization, and mortality. The percentage of acute liver injury (ALI) varied from 5 to 28%. COVID-19 hinders HCV elimination by 2030. It is recommended to continue treatment of chronic HCV and chronic HBV if already receiving treatment. Consider using antiviral therapy to prevent viral flare-ups in patients with occult or resolved HBV and COVID-19 who are receiving immunosuppressive agents. Patients with AIH do not have an increased risk of adverse outcomes even in high-risk areas. There is an association between MAFLD and disease progression. Patients with any type of cancer are at a higher risk of infection and are more likely to develop more severe clinical outcomes. Most societies advise against immunosuppressant modifications in patients with mild COVID-19, whereas in rare cases such as severe lymphopenia, worsening pneumonia, or bacterial or fungal superinfection, reduction or discontinuation of antiproliferative agents and lymphocyte-depleting therapies has been suggested.

## Introduction

Coronaviruses are enveloped single-stranded RNA diverse group of viruses infecting animals and can cause mild to severe respiratory infections in humans. A novel coronavirus designated as SARS-CoV-2 emerged in the city of Wuhan, China, causing an outbreak of viral pneumonia and spread all over the world [1, 2], named “COVID-19” [3]. The virus causes a total of approximately 437,333,859 confirmed cases and nearly 5,960,972 confirmed deaths updated on March 3, 2022 [4]. Recent reports showed that about 2–11% of patients with COVID-19 had underlying chronic liver disease and hepatic dysfunction has been seen in 14–53% of patients with COVID-19 [5].

## Pathogenesis

The pathophysiological and immunological mechanisms of liver injury in patients with COVID-19 are poorly understood.

### Hepatotropism

Angiotensin-converting enzyme 2 (ACE2), the susceptible receptor for SARS-CoV-2 virus entry into the cells, is highly expressed in the vascular endothelium of small and large arteries and veins but not in the sinusoidal endothelium, Kupffer cells, or T and B lymphocytes [5]. Chai et al. in 2020 [6] reported more abundance of ACE2 receptor in cholangiocytes (59.7%) than hepatocytes (2.6%). While ACE2 in conjunction with transmembrane serine protease2 (TMPRSS2) is considered the predominant receptor for SARS-CoV-2 entry into cells, L-SIGN (CD209L) and CD147 may function as possible alternative cell receptors for SARS-CoV-2 [7].

### Mechanisms of liver injury (Fig. 1)

#### Direct cytopathic effect

Direct hepatic infection was evidenced by showing SARS-CoV-2 particles in the cytoplasm of hepatocytes of COVID-19 patients [7, 8]. Recent evidence suggests upregulated ACE2 expression in hepatocytes, triggered by inflammatory signals, such as type I interferon or IL-6 during SARS-CoV-2 infection [9, 10]. SARS-CoV-2 directly contributed to cytopathy by conspicuous mitochondria swelling, endoplasmic reticulum dilatation, decreased glycogen granule, and impaired cell membranes in the hepatocytes [11].

**Fig. 1:**
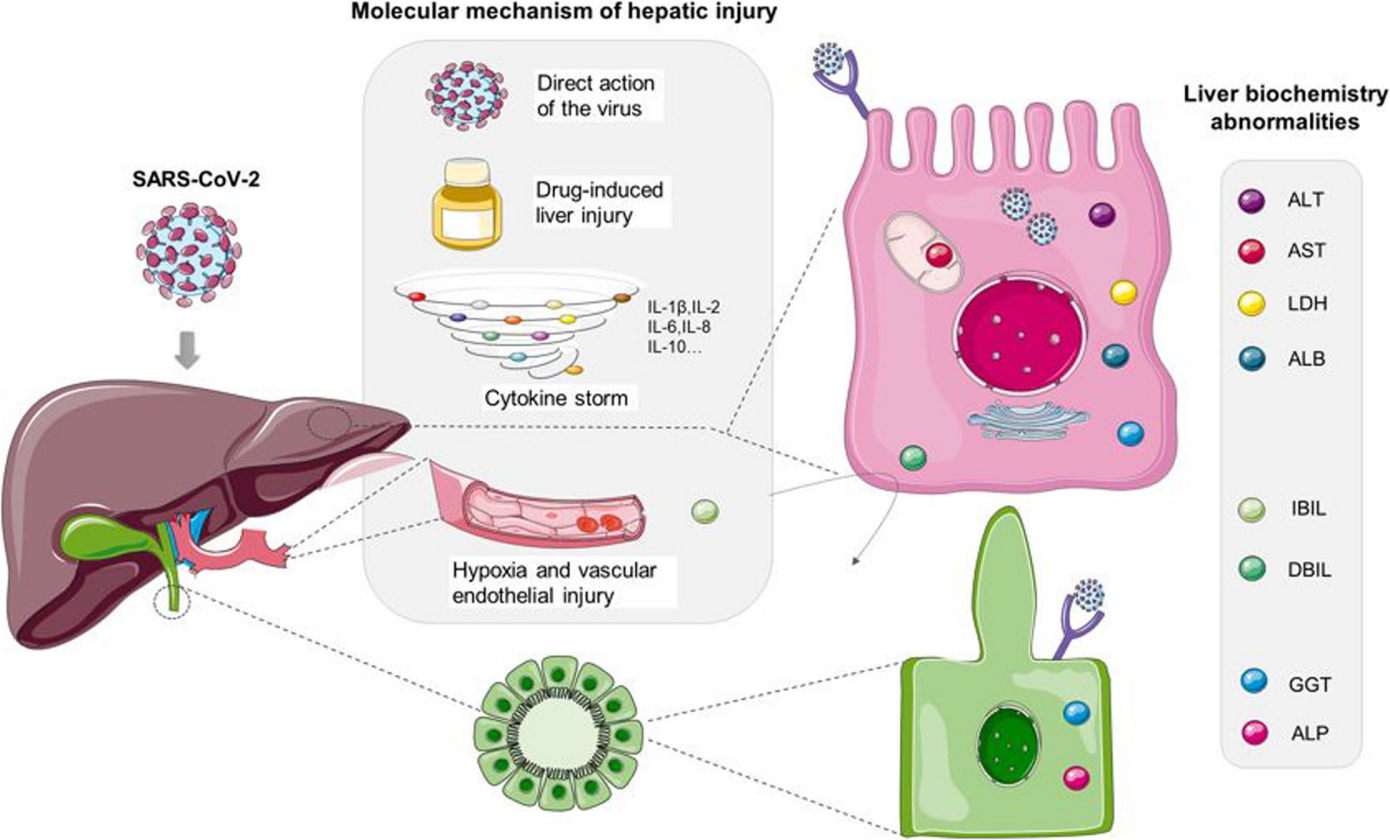
Cited in the *Mediterranean Journal of Hematology and Infectious Diseases*, *Mechanism of SARS-CoV-2 Invasion into the Liver and Hepatic Injury in Patients with COVID-19* published on January 1, 2022

#### Abnormal immune response

Crosstalk between hyperinflammation and dysregulated immune responses is characterized into three phases: the immune activation stage, secondary hemophagocytic lymphohistiocytosis (sHLH) stage, and immune suppression stage [7]. In the immune activation stage, replication of SARS-CoV-2 causes cell pyroptosis and releases of proinflammatory cytokines [12]. These inflammatory signals subsequently activate T and B cells and recruit macrophages and monocytes [13]. sHLH is a hyperinflammatory syndrome characterized by activated T cells and NK cells producing large amounts of cytokines to activate monocyte-derived macrophages [14, 15]. Activated macrophages also produce additional IL-6 and other inflammatory factors, resulting in cytokine release syndrome or *cytokine storm* that causes severe immune damage to the lungs as well as the liver [16, 17]. The immune suppression stage is characterized by a drastic reduction in peripheral lymphocytes [18, 19], T cell apoptosis, and exhaustion [7, 15].

##### Cytokine release syndrome (CRS)

IL-6 is a potential risk factor in patients with COVID-19 developing severe liver injury [20, 21]. IL-6 signals through two distinct pathways referred to as classic cis signaling or trans-signaling. and results in a systemic *cytokine storm* [22]. The trans-signaling results in CRS involving the secretion of various proinflammatory cytokines and chemokines, including additional IL-6. Thus, the feedback loop of the IL-6 amplifier (IL-6 Amp) might act as a switch to activate “cytokine storms” [15].

#### Abnormal coagulation

Abnormal coagulation has been significantly associated with poor prognosis for patients with severe COVID-19 with hepatic dysfunction. Both neutrophils and monocytes are playing a significant role in amplifying blood clotting in response to proinflammatory stimuli [23, 24].

#### Hepatic ischemia/hypoxia-reperfusion injury

Hepatic ischemia/hypoxia reperfusion injury involves a biphasic process of ischemia-induced cell injury and reperfusion-induced inflammatory response. Both ischemia and reperfusion injury lead to hepatic apoptosis and elevated liver enzymes [25, 26].

#### Drug-induced liver injury

The *mechanisms of liver injury* are variable and include dysregulated immune responses with markedly elevated plasma levels of proinflammatory cytokines as IL-6 and TNF-α [27–29], direct cytopathic damage [30], the use of antibiotics, antivirals, other traditional Chinese medicines, and secondary bacterial infection [31].

*There are liver unique concerns in the pharmacologic management of COVID-19*. Remdesivir in controlled trials demonstrated no significant impact on liver function tests as compared with placebo contrary to preclinical investigations [32]. The same conclusion came by Wang et al [33]; however, patients with advanced liver disease or with severe baseline derangements in liver biochemistry should be cautious. Tocilizumab use causes mild serum aminotransferase elevations that are self-limited and asymptomatic [34]. However, progressive jaundice requiring liver transplantation (LT) has been reported. HBV reactivation had been reported, and HBV serology should be part of the pre-treatment workup [35]. In patients with IBD, the use of corticosteroids has been associated with ICU admission, ventilator requirement, and/or death [36]. Patients treated with glucocorticoids for rheumatologic conditions have an increased rate of hospitalization following COVID-19 infection [37]. Currently, there is no need to reduce immunosuppression in patients with autoimmune hepatitis or LT recipients including the use of corticoids if required [38]. The use of anticoagulant agents demonstrated no excess of bleeding events in patients with cirrhosis and portal vein thrombosis [39]. Anticoagulation may have antifibrotic properties [40] and confer a survival advantage in patients with cirrhosis [41]. Italian multicenter study confirms this result [42].

### Liver histopathology in COVID-19

In a study done by Lagana et al. [43], *Macrovesicular steatosis* was the most common finding, involving 30 patients (75%). Mild *lobular necroinflammation* and *portal inflammation* were present in 20 cases each (50%). Vascular pathology, including *sinusoidal microthrombi*, was infrequent, seen in six cases (15%). PCR of liver tissue was positive in 11 of 20 patients tested (55%).

## COVID-19 and chronic liver disease (CLD)

Patients with CLD per se do not appear to be over-represented in cohorts of patients with COVID-19 where they make up less than 1% of reported cases [44]. Liver injury associated with SARS-CoV-2 infection is usually mild and self-limiting. Severe liver injuries correlate with a more severe clinical course reflected by higher rates of intensive care unit admission, mechanical ventilation, renal replacement therapy, and mortality [21, 45]. There is a higher risk of decompensation and failure with more reduced liver function reserve. Both hepatocellular and ductular injuries had been reported [46]. The main presentation was diarrhea or nausea/vomiting which is more likely to have severe COVID-19 [47].

Liver function test abnormalities: LFT abnormalities ranged from 37 to 69% [48–51]. The percentage of LFT abnormalities reached 80.5% in patients who died due to SARS-CoV-2 infection [52]. The percentage of ALT elevation ranged from 18.2 to 31.6%, and the percentage of AST elevation ranged from 14.8 to 35.4%. Higher percentages of total bilirubin elevation (5.1 to 11.5%) were reported in several previous studies [48, 49, 53]. The synthetic function of the liver had been affected; serum albumin was less than the normal range and prolonged prothrombin time [28]. Elevated liver enzymes, particularly AST and ALT level elevations greater than five times the upper limit of normal, are associated with an increased risk of death [21, 54, 55]. In a multi-center, observational cohort study across 21 institutions in the United States (US) of adult patients with CLD and laboratory-confirmed diagnosis of COVID-19, the overall all-cause mortality was 14.0% with 61.7% had severe COVID-19. In one of the largest studies of outcomes of SARS-CoV-2 infection in patients with CLDs with and without cirrhosis, SARS-CoV-2 infection in patients with cirrhosis was associated with 2.38 times mortality hazard, and the presence of cirrhosis among patients with CLD infected with SARS-CoV-2 was associated with 3.31 times mortality hazard [56]. In another study, collected by two international registries from the UK, 745 patients were reported from 29 countries including 386 with cirrhosis and 359 without. Mortality was 32% in patients with cirrhosis compared with 8% in those without. There was a significant increase in mortality in cirrhotic patients with CTP-B and CTP-C compared to patients without liver disease [57]. Mortality in cirrhosis increased according to Child-Turcotte-Pugh class: CTP-A 19%, CTP-B 35%, and CTP-C 51%. The main cause of death was respiratory failure (71%) in addition to advanced age, diabetes, hypertension, chronic and current smoker, and alcoholic liver disease. Hispanic ethnicity and decompensated cirrhosis were independently associated with risk for severe COVID19 [47]. The mortality rate in cirrhosis is comparable to the rates reported in smaller studies in Northern Italy (34%) [42] and North America (39%) [58].

The impact of COVID-19 extends beyond the direct morbidity and mortality associated with exposure and infection. The emergence of COVID-19 occurs at a critical moment in the context of hepatitis elimination with only 10 years remaining to reach the Global Health Sector Strategy targets by 2030 [59]. Although the full impact of delaying hepatitis elimination programs is yet to be seen. Blach et al. [60] used mathematical models to evaluate the possible impact on hepatitis disease burden and mortality resulting from programmatic delays. They reported that a 1-year delay in HCV programs could cause excess HCV morbidity and mortality. A 1-year hiatus in HCV elimination programs could result in 72,300 excess liver-related deaths and 44,800 excess liver cancers globally over the next 10 years. Most excess deaths would be in the lower-middle-income and high-income groups.

In addition to standard of care, it is recommended to continue treatment of chronic HCV and chronic HBV if already receiving treatment, use telemedicine/local laboratory testing for follow-up visits in patients receiving antiviral therapy, and send follow-up prescriptions by mail. Treatment for HCV and HBV should be initiated according to the guidelines [61, 62]; avoid interferon α, in patients with COVID-19; and postpone the initiation of treatment for HCV and HBV. Antiviral initiation should be made on a case-by-case basis if there is flare-up. Patients with occult HBV and COVID-19 receiving corticosteroids, tocilizumab, or other immunosuppressive agents should use antiviral therapy to prevent a viral flare-up. Consider early admission for all patients with cirrhosis who become infected with COVID-19 and manage these patients in the non-COVID-19 ward. Patients with new or worsening hepatic decompensation or ACLF should be prioritized for SARS-CoV-2 testing even in the absence of respiratory symptoms [63]. All cirrhotic patients should receive vaccination for *Streptococcus* pneumonia and influenza [38].

### COVID-19 and acute liver failure

The percentage of acute liver injury (ALI) varied from 5 to 28%. This wide range of differences in the percentage of ALI might be due to differences in the percentage of patients with severe COVID-19 or differences in the percentage of patients with underlying chronic liver diseases [48, 50]. Ji et al. [46] reported studying 140 consecutive COVID-19 patients with pre-existing CLD. They had liver cirrhosis, NAFLD, chronic HBV, and chronic HCV infection. Only one patient with CLD had ACLF. On the day of death, IL-6 and serum ferritin levels increased rapidly reaching the highest levels favoring immune-mediated attacks. Hypoxic hepatitis was another possible cause that was reported in 2.5% of ICU patients [64]. Another study reported acute hepatic decompensation occurred in 179 (46%) of patients with cirrhosis of which 21% had no respiratory symptoms; 89 (50%) of those with hepatic decompensation had ACLF [65].

#### Autoimmune liver disease (AIH)

Several reviews endorse that even in fairly endemic areas, sufferers with AIH are not displaying extended hazards of detrimental consequences. In a study from Italy, 26% of AIH patients reported COVID-19 that did not require hospitalization. Only 4 confirmed cases, 3 of which were hospitalized and 1 old-aged patient with comorbidities, died [66]. No difference between AIH and non-AIH CLD regarding hospitalization, ICU admission, and/or death in a recent international study involving 70 patients with AIH [67]. No justification for the hypothesis that immunosuppressed patients are more vulnerable to SARS-CoV-2 infection compared to the general population [68]. This supports the idea that immunosuppressive treatment should not be stopped in patients with AIH, but preventive measures remain crucial. The EASL and AASLD recommend the continuation of immunosuppressive regimens without any dose modification in patients without COVID-19. To avoid exposure to high doses of corticosteroids, it is recommended to use budesonide as a first-line agent to achieve remission in non-cirrhotic patients with exacerbation of AIH [38]. This is contrary to patients with AIH who become infected with COVID-19. According to the EASL recommendations, switching to dexamethasone or adding it to the basic corticosteroid should be an option only for patients with severe disease. According to the AASLD, corticosteroids as well as azathioprine or mycophenolate mofetil should be reduced to the lowest possible doses, particularly if the course of COVID-19 is severe [69]. Vaccination for *Streptococcus* pneumonia and influenza is essential for all patients [38].

#### Non-alcoholic fatty liver disease (NAFLD)

Obesity represents a great hazard for severe COVID-19 [70]. Hu and his colleagues [71] reported in a study of 58 patients with overweight/obesity and/or abnormal liver function with COVID-19 that obesity is a predisposing factor for prolonged hospitalization in patients with COVID-19 infection. Adipose tissue may serve both as a viral reservoir and also as an immunologic hub for the inflammatory response [72]. Recently, Meijnikman et al. [73] reported that ACE2 is upregulated in the subcutaneous and visceral adipose tissues and liver tissue of individuals with NAFLD; fostering viral penetration into cells identified an additional mechanism that contributes to increased susceptibility to severe COVID-19 in individuals with NAFLD. There is an association between NAFLD and disease progression in a retrospective cohort of 202 patients with COVID [74]. This additional risk has been observed even in younger patients with NAFLD [75] and in the absence of type 2 diabetes mellitus [76]. Within patients with NAFLD, non-invasive fibrosis scores correlate with a better probability of developing severe COVID-19 illness regardless of metabolic comorbidities [77].

#### Hepatocellular carcinoma (HCC)

Zhang et al. [78] reported that patients with malignancy had poorer outcomes when compared to the general population. They studied 28 cancer patients; 2 of them had HCC. Age of the patients, associated comorbidities, and underlying cirrhosis were risk factors. Anemia and hypoproteinemia affect nutritional status, which compromises their immunity, making them more vulnerable to severe infection. Co-infection with COVID-19 with cancer increased vulnerability to severe disease and increased chance of mortality, as well as ICU admission. Iavarone et al. [79] recommended the use of telemedicine to decrease the risk of the spread of COVID-19. Indications of liver transplantation (LT) and locoregional therapy (LRT) in HCC patients have not changed. It is better to reserve LT for highly progressive cases because of the shortage of ICU beds and to use LRT as a salvage procedure to decrease the risk of HCC progression during the waiting period. Boettler et al. [80] agreed that stereotactic body radiation therapy (SBRT) is another alternative ablative option that can be offered to patients eligible for TACE, especially those with lesions near vascular structures, whom their procedures have been canceled because of COVID-19 [81]. Continuing surveillance imaging of HCC patients with an acceptable delay of a maximum of 2 months is recommended [69]. Local ablative therapies should not be delayed for eligible patients. Post-embolization syndrome can be treated by corticosteroids to minimize hospital stay even in patients proven to have COVID-19 except if there is any other contraindication. TACE is better than SBRT as it can be separated by intervals ranging from 4 to 12 weeks, according to the response and characteristics of the lesion. For patients with resectable HCC whose surgical procedures have been canceled, TACE can be used as a bridge to definitive treatment [82]. Sorafenib is recommended to be continued without any change in its dose [83]. Nivolumab might be temporarily suspended to avoid exposure to COVID-19 at the infusion center [84]. However, Andersen et al. [85] in a retrospective study reported that chronic use of immunosuppressive drugs was neither associated with worse nor better clinical outcomes among adults hospitalized with COVID-19.

#### Liver transplantation (LT)

In one of the largest multicenter network studies on LT and COVID-19, Mansoor et al. [86] found LT patients with COVID-19 to have a significantly higher risk of hospitalization but not mortality, thrombosis, or ICU requirement compared to patients without LT and COVID-19. LT reduced even in areas with a low prevalence of COVID-19 [87]. Deceased organs infected with SARS-CoV-2 even mild or asymptomatic infections are no longer used [88]. However, few recommend the usage of COVID-19-positive liver donors in very selected cases [89]. Donor testing should be proofed preferentially by bronchoalveolar lavage in deceased donors or by nasopharyngeal swab in living donors [88]. Liver grafts recovered from COVID-19-positive deceased donors 3–4 weeks after symptom resolution and 2 negative swabs 1 day apart are considered safe for transplantation, otherwise, deferred [90]. It is mandatory to test living donors at least once within 1–3 days before transplantation. It is recommended to do self or hospital-based quarantine for 2–3 weeks with preventive transmission counseling. COVID-19-positive or high-risk living donors should postpone donation until at least 3–4 weeks after symptom resolution and until 2 negative PCR tests have been observed [90]. The recipient should undergo a PCR test within 1 day of transplantation. Recipients with clinical suspicion or active SARS-CoV-2 infection should have transplantation deferred until 4 weeks after symptom resolution and after 2 negative tests at least 1 day apart [88]. Urgent transplantations would proceed first with temporary cessation of elective living donor and non-urgent deceased donor LT during the pandemic [88]. According to the MELD score, risk of dropping out particularly from liver cancer progression, and fulminant hepatic failure, elective cases could be phased later [90].

No great difference in mortality was found in LT patients with COVID-19 whether mild or severe in comparison with the general population. The incidence in a large Italian LT survey was 1.25%, and most patients (75%) had a mild disease [91]. Conversely, a worse outcome was recorded in New York City [92].

In a systematic review, liver transplant patients with confirmed COVID-19 from 15 studies, were more likely to present with concurrent diarrhea [93]. In a nationwide Spanish study, chronic use of immunosuppressive agents did not increase mortality rates; however, at doses higher than 1 mg/day mycophenolate increases the risk of severe COVID-19 among hospitalized LT patients [94]. Mycophenolate produces a cytostatic effect on activated lymphocytes [95]. Mycophenolate and SARS-CoV-2 exert a synergic and deleterious effect on depleting peripheral lymphocytes [96]. Calcineurin inhibitors not only have antiviral effects but also can ameliorate the cytokine storm [97]. It is better to continue the medication even after the COVID-19 diagnosis. However, consider dose reduction or temporary conversion to calcineurin inhibitors or everolimus until complete recovery from COVID-19 in patients receiving mycophenolate [94].

In patients with mild COVID-19, no need to make immunosuppressant modifications. In case of severe lymphopenia, worsening pneumonia, or bacterial or fungal superinfection, reduction or discontinuation of antiproliferative agents and lymphocyte-depleting therapies has been suggested [87].

#### COVID-19 vaccination in patients with liver diseases and liver transplant recipients

The already licensed COVID-19 vaccines are immunogenic, and the short-term safety record appears excellent in healthy individuals aged 16 years. Based on current knowledge, there is no evidence to contradict the safety and immunogenicity of currently approved vaccines in patients with CLD and hepatobiliary cancer or in immunocompromised patients after liver transplantation. Given the high risk of serious health consequences of SARSCoV-2 infection in such patients, the potential benefits of the vaccine are likely to outweigh the risks associated with vaccination. Therefore, SARS-CoV-2 vaccination is recommended in patients with CLD, hepatobiliary cancer, and candidates for liver transplantation. Optimal timing of vaccination in transplanted recipients is still unestablished but vaccination 3–6 months after transplantation is advisable [98]. Recently, John and his colleagues reported that two doses of a COVID-19 mRNA vaccine are associated with a decrease in COVID-19 infection and death in liver transplant recipients [99].

## Data Availability

NA.

## Abbreviations

ACE2: Angiotensin-converting enzyme 2
sHLH: Secondary hemophagocytic lymphohistiocytosis
NK cells: Natural killer cells
IL-6: Interleukin-6
CRS: Cytokine release syndrome
sIL-6R: Soluble IL-6Receptor
IL-6 Amp: IL-6 amplifier
NETs: Neutrophil extracellular traps
LFT: Liver function tests
ALI: Acute liver injury
CLD: Chronic liver disease
ACLF: Acute on top of chronic liver disease
NAFLD: Non-alcoholic fatty liver disease
CTP: Child-Turcotte-Pugh class
HCC: Hepatocellular carcinoma
ALD: Alcohol-related liver disease
AIH: Autoimmune hepatitis
AASLD: American Association of Study of Liver Disease
LRT: Locoregional therapy
SBRT: Stereotactic body radiation therapy
TACE: Transarterial chemoembolization
